# Laparoscopic Diagnosis of Incarcerated “Spigelian Hernia”: Report of a Case and Review of the Literature

**DOI:** 10.1155/2011/491802

**Published:** 2011-10-19

**Authors:** Sanoop Koshy Zachariah, Priya Jose

**Affiliations:** Department of General Gastrointestinal and Laparoscopic Surgery, Medical College, Kolenchery, Cochin, Kerala 682311, India

## Abstract

Spigelian hernias are rare hernias, representing only about 1-2% of all abdominal hernias. An accurate preoperative diagnosis of this condition is often difficult because the physical presence of these hernias is often not demonstrable owing to its peculiar anatomic location. Many patients with Spigelian hernias, therefore, may have no obvious findings on clinical examination. The diagnosis is even trickier in obese patients wherein the hernia sac may lie in an intraparietal plane, masked by the abdominal subcutaneous fat. Here we describe a case of incarcerated Spigelian hernia where clinical and radiological findings were inconsistent and the accurate diagnosis was made by laparoscopy and was followed by a laparoscopic-assisted repair using an open anterior approach with an onlay mesh. We therefore feel that laparoscopy can be a useful tool for confirming the diagnosis and also for performing a definitive repair in doubtful cases of Spigelian hernias.

## 1. Introduction

The Spigelian hernia (SH) is named after the Belgian anatomist Adriaen Van Den Spigehel who described the semilunar line (linea spigeli) in 1645 [1]. However, the Spigelian hernia was first described and defined by Josef Klinkosch in 1764. [2]. A Spigelian hernia is the protrusion of preperitoneal fat, peritoneal sac, or viscera through a congenital or acquired defect in the Spigelian aponeurosis. These are rare hernias representing only about 1-2% of all abdominal hernias and fewer than 1,000 cases being reported in the literature. Although the incidence of Spigelian hernias is low, they are the most common type of spontaneous lateral ventral hernias. They frequently present between 50–60 years of age, with a male to female ratio of 1 : 1.18 and occur twice as often on the right side compared to the left [2]. Reported incarceration rates are around 17%, with acute presentations in 10% and the lack of reliable physical examination findings in 36% of patients [3].

## 2. Case Report

A 45-year-old-woman presented with a history of sudden onset of right-sided lower abdominal pain since five hours and was associated with vomiting. She gave a history of recurrent episodes of right-sided lower abdominal pain since the past 2 years and several imaging studies done over this time period, including ultrasonography and computed tomography scans, failed to reveal the etiology of her symptoms. She had no previous surgical history, but was recently detected to have type 2 Diabetes Mellitus. Clinical examination revealed an obese abdomen with maximal focal tenderness in the right lower quadrant. However, a vague fullness was palpable in the lower right quadrant which corresponded to the site of maximal tenderness. She was afebrile, the heart rate was 90/minute, blood pressure was 140/90 mm of Hg, and the bowel sounds were hyperperistaltic. Laboratory investigations were unremarkable except for neutrophilic leucocytosis. Plain abdominal radiograph and ultrasonography were inconclusive. Hence, with a provisional clinical diagnosis of an appendicular pathology, the patient was taken-up for laparoscopic surgery.

**Figure 1:**
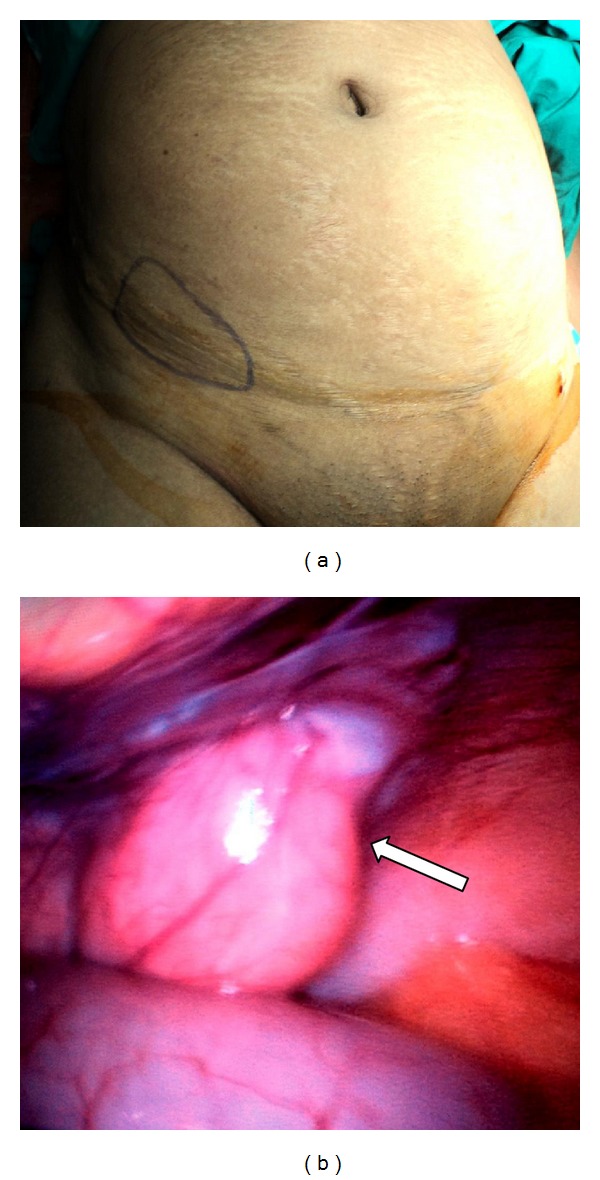
(a) Area of tenderness marked out on the abdominal wall prior to induction of anaesthesia. (b) Laparoscopic view of the Spigelian hernia.

**Figure 2:**
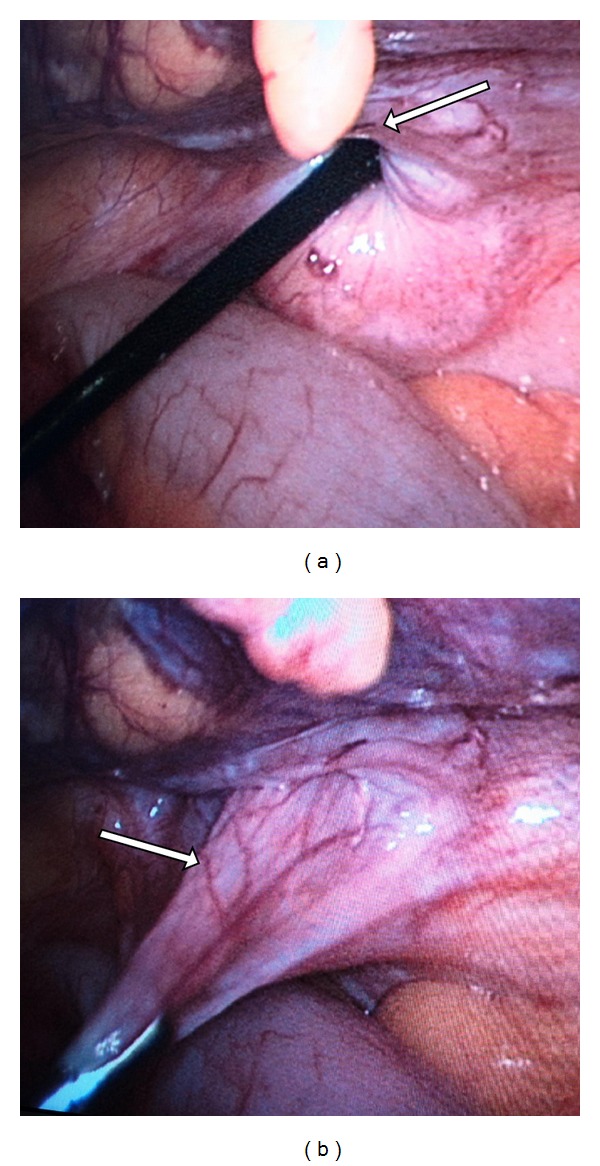
(a) The laparoscopic grasper tool demonstrating the slit like “Spigelian defect”. (b) The hernia sac (arrow) after laparoscopic reduction of the incarcerated bowel.

**Figure 3:**
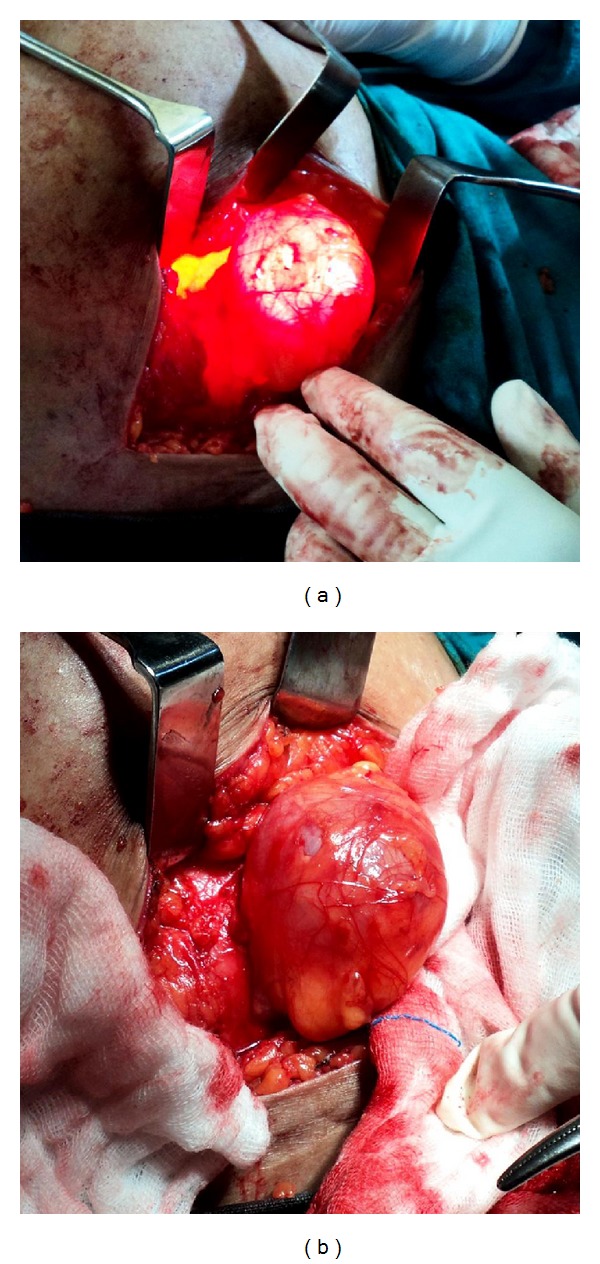
The external view of Spigelian hernia sac with (a) and without (b) transillumination with the laparoscope's light source.

Prior to the induction of anaesthesia, the patient's abdomen was palpated and the area of maximal focal tenderness and the fullness was outlined with a marking pen (Figure 1(a)). Pneumoperitoneum was created by an open technique and a 30-degree 10 mm telescope was used for the procedure. Immediately upon entry, a loop of small bowel was seen herniating through a defect in the anterior abdominal wall and this coincided with the area that was marked over the skin (Figure 1(b)). This site was in the territory of the “Spigelian hernia belt” and hence consistent with a Spigelian hernia. An additional 5 mm port was placed in the left iliac fossa for the grasper tool. Laparoscopic reduction of bowel was done and a slit-like Spigelian defect about 3 cm by 2 cm was clearly seen (Figures 2(a) and 2(b)). The incarcerated bowel which was the mid ileum appeared viable and peristalsis was noted. Now as the diagnosis was certain and as the patient could not afford a composite dual mesh, we decided to do a conventional hernia repair using a regular polypropylene mesh instead of a totally laparoscopic repair. Thus, a transverse incision was made over the site of the hernia on the anterior abdominal wall. The sac was identified below the arcuate line which illuminated well with the light transmitted from the laparoscope (Figures 3(a) and 3(b)). The sac was excised and defect was repaired using an onlay polypropylene mesh as for an open hernioplasty. The repair was then inspected from within with the help of the laparoscope and found to be adequate, and there was no bulge at the repaired site even on air-insufflation of the peritoneal cavity. Postoperatively, the patient made good progress and was discharged on the seventh postoperative day. The patient is on regular follow-up since 6 months and is doing well.

## 3. Discussion

The exact etiology of Spigelian hernia is uncertain. It could be either congenital or acquired [4]. The congenital theory suggests that the Spigelian fascia is weakened by the presence of perforating vessels which predispose to herniation. Acquired causes suggest weakening of the abdominal wall due to collagen disorders, aging, obesity, rapid weight loss, multiple pregnancies, trauma, and surgery. The contents commonly include preperitoneal fat, omentum, bowel, and occasionally other organs.

The accurate diagnosis of SH is often difficult and can be attributed to its rarity and the absence of classical symptoms coupled with a lack of personal clinical familiarity. It can simulate a variety of other commoner lower-quadrant abdominal diseases. Only about 50% of cases are diagnosed preoperatively [5, 6]. The anatomical location contributes to the diagnostic difficulty. It occurs through a defect in the Spigelian fascia, that is, the transversus abdominus aponeurosis lateral to the rectus muscle, often at the level of the arcuate line, where the fascia is widest and weakest. The hernial sac may then expand and traverse primarily through an interparietal plane, laterally and caudally between the external and internal oblique muscles, with no obvious mass on inspection or palpation. Therefore, patients with Spigelian hernias may have no obvious finding on physical examination. The commonest presentation is that of a painful lump lateral to the rectus muscle, below the level of the umbilicus. This line is referred to as the Spigelian line. More than 50% of all Spigelian hernias are found in a 6 cm wide region inferior to the umbilicus and superior to the inferior epigastric vessels. This region is referred to as “the Spigelian hernia belt” [7]. Large SH, in thinner individuals are usually easy to identify when compared to the small hernias associated with smaller abdominal wall defects that can be easily missed as they are often masked by subcutaneous fat and an apparently intact aponeurosis. In uncomplicated cases, the symptoms may be intermittent, making diagnosis more often than not very ambiguous. As with all hernias, there is a risk of strangulation, and it is a rare cause of the acute abdomen [8].

Radiological investigations, such as ultrasonography and Computed Tomography (CT) scans may help diagnose incarcerated Spigelian hernias. Papierniak et al. have demonstrated the usefulness of computerized tomography in diagnosing Spigelian hernias [9]. Yet in uncomplicated cases, a clear radiologic diagnosis of an SH prior to surgery is often infrequent.

Spigelian hernias have conventionally been repaired with an open surgical technique. Zacharakis et al. [10] have shown that the technique of plug and mesh repair is a safe and effective method of repairing an incarcerated Spigelian hernia. Minimally invasive techniques are becoming more and more popular. Laparoscopy has been shown to be a useful tool for the confirmation of diagnosis and also for performing a definitive repair [11–14]. In 1992, Carter published the first intraabdominal laparoscopic repair of SH [15]. Morena-Egea et al. [16] described the procedure of total extraperitoneal laparoscopic approach for SH. Laparoscopic repair of SH has been shown to offer certain advantages over open-mesh repair, in terms of lower morbidity, shorter hospital stay, better cosmesis, and perhaps a lower recurrence rate.

In our case, the diagnosis of SH was made laparoscopically. The clinical examination revealed only a tender area with a vague fullness and no palpable mass. This was probably because the hernial sac was small and was masked by an apron of subcutaneous fat. Moreover, as the hernia was incarcerated, there was no demonstrable expansile impulse on coughing. The defect could be clearly visualised with the help of the laparoscope and the sac could also be identified intraabdominally and isolated extraperitoneally. The hernioplasty was done by an anterior approach using a polypropylene mesh similar to an open technique and was therefore cost-effective. The immediate integrity and adequacy of the repair could be checked laparoscopically after insufflating the peritoneal cavity with air. However, we do feel that some of the reported benefits of a totally laparoscopic hernia repair could not be obtained in this case.

In conclusion, the diagnosis of Spigelian hernias is not always straightforward, especially when a mass is not palpable. Laparoscopy can be a useful tool to confirm the diagnosis of Spigelian hernias especially when clinical and radiological findings may be equivocal. Laparoscopy can help delineate the exact site of the defect in the anterior abdominal wall and also help identify the sac. A laparoscopic-assisted technique, like in this case, may be cost-effective especially in rural hospitals.

##  Disclosure

This paper has not been published in its current form or a substantially similar form (in print or electronically, including on a website), that it has not been accepted for publication by another journal and that it is not under consideration by another publication. One of the authors hereby transfers, assigns, or otherwise conveys all copyright ownership, including any and all rights incidental thereto, exclusively to the journal, in the event that such work is published by the journal. 

##  Conflict of Interests

The authors have no conflict of interests. 

##  Consent

The patients consent has been obtained for publication. 

##  Authors' Contribution

The entire paper was conceived, designed, and drafted by Dr. Sanoop K Z, and Dr. Priya J was involved in revision and drafting the final paper.
